# Political construction of risk perception and preventive behaviour during the COVID-19 pandemic in the Republic of Korea

**DOI:** 10.7189/jogh.15.04189

**Published:** 2025-07-04

**Authors:** Seunghoo Jeong, Ji-Bum Chung, Minjun Kim, Min-Kyu Kim

**Affiliations:** 1 Track & Civil Infrastructure Division, Korea Railroad Research Institute, Uiwang, Republic of Korea; 2 Department of Civil, Urban, Earth, and Environmental Engineering, Ulsan National Institute of Science and Technology, Ulsan, Republic of Korea; 3 Department of Urban Engineering, Chungbuk National University, Cheongju, Republic of Korea

## Abstract

**Background:**

The COVID-19 pandemic has highlighted the complex interactions between politics and public health, as political ideologies shape risk perception and adherence to safety measures worldwide. In South Korea, the shift from a progressive to a conservative government administration during the pandemic presents a unique opportunity to examine how risk perception and compliance with preventive measures vary under different political regimes.

**Methods:**

We used secondary data from a representative South Korean polling company. Biweekly repeated cross-sectional surveys were conducted 80 times over three years (February 2020–April 2023), capturing citizens’ perceptions during the regime change. We analysed time-series trends in risk perception and preventive intentions. We conducted cross-sectional analyses on surveys collected under the two administrations to explore how political affiliation influenced risk perception and behaviour. These analyses provide insights into the interaction between political alignment and individuals’ risk perceptions and preventive actions.

**Results:**

Korean citizens who shared the same political views as the central government tended to trust the government’s handling of the pandemic more. As a result, they perceived less risk and engaged in fewer preventive behaviours. Rather than changes in specific quarantine policy, Korea’s case represents intergroup polarisation driven by political affiliation. We revealed compelling evidence of political influence on risk perception and behaviour, offering valuable insights for addressing politicised issues in future pandemics.

**Conclusions:**

The findings of this study suggest that COVID-19 has become a political issue not only between two hostile political parties but also among individuals with different political preferences. Therefore, when striving for collaborative problem-solving, caution must be exercised against politicising the issues as witnessed during the COVID-19 pandemic. These politicised subjects may result in criticising the other party through prejudicial criticism, as well as refraining from making efforts to find common values.

Risks that are perceived as dreadful, uncontrollable, and unfamiliar tend to be regarded as dangerous, particularly when compared with other existing and anticipative risks [1,2]. Such perceptions of a specific risk can be amplified or diminished, influenced by factors such as hazard characteristics [1,2]. In addition to hazard characteristics, receiver traits can also significantly influence the severity of risk [2]. Risk perception can vary among individuals based on not only their demographic traits, psychological aspects, value orientation, and the levels of domain-specific knowledge related to the particular risk [2]. For example, Japanese citizens who experienced the Fukushima nuclear disaster may have had significantly greater concerns about nuclear energy than those from countries without such direct exposure. Similarly, Koreans who have witnessed the severe impact of the Middle East respiratory syndrome outbreak in their country may perceive the risk of COVID-19 more intensely than citizens from other countries. Therefore, individuals’ risk perception can be socially constructed and often varies depending on the perceivers’ traits and the surrounding circumstances [3,4].

Generally, the studies on risk perception can be divided mainly into realist and constructionist viewpoints [3,4]. The realist viewpoint assumes that risks are tangible by-products of natural and social processes, and can be objectively mapped, measured, and controlled. It prioritises experts’ opinions over those of laypersons and identifies policy implications by utilising quantitative research methods based on psychometric measurements of individuals [3–6]. By contrast, the constructionist viewpoint assumes that risks can be socially constructed and are affected by many social and cultural factors. It stresses the importance of public participation of social and interest groups, as well as processes such as public debates, negotiation, and conflict resolution [4,7,8]. The constructivist perspective underscores how the values and cultural pressures of diverse social groups significantly influence individuals and shape their risk perceptions.

From this constructivist perspective, it becomes apparent that for some risks, collectively shared political biases can exert a significant impact on individuals’ risk perception. A representative example of the constructivist risk phenomenon is the risk perception associated with nuclear energy. Politically conservative individuals across various countries and cultures tend to favour nuclear energy, often resulting in lower levels of perceived risk [9–12]. This preference appears to be rooted in the cultural perception of nuclear technology as a symbol of national power and as a catalyst for development within politically conservative circles [13]. However, political conservatives generally show less support for emerging technologies such as nanotechnology, stem cell research, and agricultural biotechnology, potentially influenced by their religious beliefs [14]. As such, a shared political ideology can impact how individuals perceive potential risks associated with these technologies.

Similar to the political controversies surrounding nuclear energy, recent research has shown that political orientation significantly shapes COVID-19 risk perceptions and policy preferences [15–22]. A representative example can be found in the USA during the early stages of the pandemic, when Republican government officials downplayed the severity of COVID-19 by comparing it to seasonal influenza. During this period, Democrats perceived higher risks of infection, hospitalisation, and death compared to Republicans, and were more supportive of maintaining restrictive policies such as mask mandates and social distancing guidelines [16]. Political beliefs were also found to influence trust in government; individuals with higher levels of trust were more likely to follow public health guidance [20]. Beyond the USA, survey data in South Korea shortly after the onset of COVID-19 showed that conservatives reported higher levels of risk perception than liberals during the presidency of the progressive administration [19]. Research in Argentina also provided clear evidence that political orientation was the primary factor associated with acceptance of restrictive government measures [21].

Although previous studies have offered valuable insights into the polarisation of COVID-19 risk perception, several limitations remain. COVID-19, as a new, unfamiliar, and frightening virus, led to a socially amplified perception of risk among individuals depending on their political inclinations [23]. However, much of the existing evidence is derived from relatively short-term or cross-sectional surveys, which may fail to capture the dynamic evolution of public risk perception throughout the pandemic. Accordingly, it is essential to investigate how risk perceptions have changed over the full duration of the pandemic. Moreover, few studies have sufficiently addressed the potential influence of political regime changes. Throughout the pandemic, South Korea experienced a change in government from a progressive to a conservative administration on 10 May 2022. Such a transition to a ruling party with an opposing political stance may significantly reshape trust in government, the dissemination of information, and compliance with public health directives over time. Importantly, individuals who hold political views contrary to those of the new administration may revise their evaluations of government credibility and associated risks. Consequently, politically charged issues related to the pandemic may result in divergent threat perceptions and policy preferences based on individuals’ political orientations [22].

In this study, we aimed to investigate whether individuals’ risk perception and intention to engage in preventive behaviour were politically constructed based on their political orientations during the COVID-19 pandemic. Specifically, we focused on examining how citizens perceive COVID-19 risk differently under two government regimes with distinct political stances in the Republic of Korea. We utilised biweekly repeated cross-sectional surveys, conducted 80 times during the pandemic over a three-year period (February 2020–April 2023). The survey data were sufficient for monitoring changes in individuals’ perspectives on COVID-19 amid the transition of Korea’s government regime from a progressive to conservative orientation. We conducted both longitudinal and cross-sectional analyses to uncover an intergroup polarisation in COVID-19 risk perception. We illustrated temporal trends in risk perception and preventive behaviour among individuals with different political orientations. We conducted cross-sectional analyses using partial least squares structural equation modelling (PLS-SEM) to investigate whether objective risk or subjective political inclination influences individuals’ risk perception and their preventive behaviour. These two methods can provide insights into how the political alignment of South Korean citizens with the government influences their attitudes towards the pandemic.

The Republic of Korea has a firmly established two-party system. As various issues become politicised, conflicts and defences between political parties intensify. The media also plays a significant role in this matter, generating numerous controversies, while individuals form their own perceptions and attitudes influenced by the prevailing social and political atmosphere [23]. Jasanoff noted that risk perception is inherently political [4]. Accordingly, COVID-19 risk perceptions among Koreans can be constructed differently based on their political biases. The emergence of the new coronavirus disease and its subsequent politicisation under two distinct political administrations have motivated this study.

According to Sachs et al. [24], Korea’s index of epidemic control, which combines the mortality rate, effective reproduction rate, and efficiency of epidemic control, surpasses that of other Organisation for Economic Co-operation and Development (OECD) nations. Following several mass infection outbreaks in Korea, the central government declared the highest national alert level on 23 February 2020 [15], and subsequently launched the ‘Strengthened Social Distance Campaign’ on 22 March 2020 [25]. The social distancing policy was sufficiently effective in significantly reducing public activities without the implementation of lockdown measures. Due to stringent government policies and widespread public compliance with measures such as mask-wearing, the Korean government successfully contained the infection until the emergence of the Omicron variant [15]. As the Omicron mutation began spreading in Korea on 1 December 2021, tens of thousands of confirmed cases emerged daily. However, as the number of confirmed cases increased, people began to perceive COVID-19 as a more familiar risk rather than a dreadful and unfamiliar one. This has led many people to become less sensitive to the COVID-19 risk, ultimately resulting in fatigue with the government’s long-term quarantine policy [26].

Unlike other countries, the South Korean government decided against banning Chinese individuals from entering the country when the COVID-19 infection first occurred in Wuhan, China. This decision faced strong criticism from the conservative People Power Party, the opposition party at the time, sparking a political debate. Despite the initial effectiveness of measures to control the spread of COVID-19, intense political debates continued to surround Korea’s COVID-19 policies, and a sharp political divide emerged from the public’s response to them. Individuals aligned with the politically progressive ruling party regarded adherence to COVID-19 policies as a means of supporting the government. Conversely, conservatives criticised these policies, arguing that excessive government interventions could lead to economic losses and violations of civil liberties [27]. COVID-19 remained a highly sensitive political issue even as the political regime eventually shifted from the progressive Democratic Party of Korea to the conservative People Power Party after the new presidential inauguration on 10 May 2022.

## METHODS

### Data explanation

To investigate how Korean people perceived the COVID-19 pandemic, biweekly survey data were collected over 80 times spanning from February 2020 to April 2023. The 80 survey data sets can be divided into two periods. The 1st–56th surveys were collected during the first period, which was characterised by a progressive-oriented administration led by President Moon Jae-in. Thereafter, until the 80th survey marks the second period, which was the conservative administration led by President Yoon Suk-yeol. Each survey comprised 1000 respondents aged ≥19 years, selected through multi-stage stratified random sampling based on sex, age, and the 17 metropolitan regions in Korea. The total sample included 80 000 participants who completed at least one of the biweekly questionnaires throughout 80 surveys. The participants were recruited from the online survey panels of Hankook Research, a leading Korean polling company. Given the difficulty of retaining the same individuals over an extended period, the collected survey responses were treated as pseudo-panel data, which are widely used in such contexts to capture population-level trends. This approach is particularly suitable for examining public opinion during prolonged events such as the COVID-19 pandemic. Pseudo-panel methods have been frequently applied in previous research when longitudinal panel data are unavailable [28,29].

We selected a total of seven variables from survey questionnaires regarding political affiliation, government trust, risk perception, and intention to engage in preventive behaviour (**Table 1**). We assessed the political orientation of each respondent using the following question: ‘How would you describe your ideological orientation?’ Responses were evaluated on an 11-point Likert scale (0 – very progressive stance and 10 – a very conservative viewpoint). We evaluated the government trust using two questionnaires, specifically trust in the presidential office and satisfaction with the government’s COVID-19 response. Both questions were reverse-coded for enhanced interpretability as follows. The response to the trust question ‘To what extent do you presently trust the Office of the President in managing the COVID-19 response?’ was evaluated on a four-point Likert scale (1 – not confident at all and 4 – very confident). Satisfaction with the government’s response to COVID-19 was assessed by the question ‘How well do you think the central government is responding to the COVID-19 pandemic?’ on a four-point Likert scale (1 – doing very poor and 4 – doing very well).

**Table 1:** Variables used in the longitudinal and cross-sectional analyses

Variable	Explanation	Source
Political orientation	Political orientation was assessed on an 11-point Likert scale (0 = a very progressive stance; 10 = a very conservative stance)	Survey (Hankook research)
Satisfaction	Satisfaction with the central government response to COVID-19 was assessed on a 4-point Likert scale (1 = doing very poor; 4 = doing very well)	
Trust	Trust in the Office of President managing the COVID-19 was assessed on a 4-point Likert scale (1 = not confident at all; 4 = very confident)	
Severity	The perceived severity of the COVID-19 pandemic in Korea was assessed on a 5-point Likert scale (1 = not at all serious; 5 = very serious)	
Infection possibility	Possibility of COVID-19 infection was assessed individually on a 5-point Likert scale (1 = not at all; 5 = to a great extent)	
Refrain from going out	Refrain from going out compared to before COVID-19 (1 = not at all; 4 = extremely)	
Refrain from eating out	Refrain from eating out compared to before COVID-19 (1 = not at all; 4 = extremely)	
COVID-19 infection	Natural logarithm of the mean value for confirmed COVID-19 cases during 7 d preceding each survey	The Ministry of Health and Welfare, Korea
Social distancing level	Social distancing level reported by the Ministry of Health and Welfare in Korea government	

COVID-19 – coronavirus disease 2019

Based on previous studies examining people’s risk perceptions of COVID-19, risk perception was assessed in terms of severity and infection possibility [30,31]. Severity was evaluated through the question ‘How seriously do you perceive the COVID-19 pandemic to be in Korea?’ using a five-point Likert scale. The infection possibility was measured using the question ‘How likely do you think you are to be infected with COVID-19?’ on a five-point Likert scale. Two questions were asked to determine the intention to engage in preventive behaviour; one regarding the extent of refraining from going out and another concerning the extent of refraining from eating out compared with the pre-COVID-19 period. Respondents responded on a four-point Likert scale. A higher score meant increased restraint in both going out and eating out, indicating a more preventive behaviour.

In addition to the survey results, we collected data relevant to the COVID-19 pandemic, encompassing confirmed infection cases and governmental policy (**Table 1**). The Ministry of Health and Welfare of South Korea has officially archived the daily confirmed COVID-19 cases. Building upon the findings of a prior study [32], we calculated the average number of confirmed cases for the seven days preceding each survey within the study time frame. To account for the exponential rise in confirmed cases following the prevalence of the Omicron variant, COVID-19 infection was assessed by applying the natural logarithm of the seven-day average cases, yielding 80 data points for the survey period. It is noted that the phenomenon of psychic numbing was observed in South Korea during the COVID-19 pandemic [26]. Initially, individuals received a heightened level of risk perception despite a small number of confirmed cases. However, the persistent threat eventually resulted in a diminished sensitivity to the pandemic, even amid an exponential increase in infections. A previous study revealed that individuals’ risk perception is closely associated with the natural logarithm rather than the absolute number of confirmed COVID-19 cases, providing clear evidence of psychic numbing. Hence, we utilised the COVID-19 infection variable with the natural logarithm value.

The social distancing level was derived from official COVID-19 reports provided by the Ministry of Health and Welfare [33]. In the early stage of the pandemic, the Korean government proactively introduced a comprehensive array of social distancing measures, including school closures and the postponement, cancellation, or downsizing of large group meetings. The initial phases of the social distancing campaign comprised three distinct stages: enhanced social distancing (22 March–19 April 2020), eased social distancing (20 April–5 May 2020), and a transition to distancing in daily life (6 May–22 August 2020). However, these initial phases were criticised for lacking systematic categorisation [34]. In response, the Korean government implemented a more structured five-level scheme, encompassing levels one (least severe) to three (most severe), with intermediate levels of 1.5, two, and 2.5. In July 2021, a new four-level framework was introduced, ranging from level one to four. The Omicron variant has since subsided, and all restrictions were lifted on 18 April 2022. Within the study period, each of the 80 survey time points can be assigned a specific social distancing level.

### Cross-sectional analysis (PLS-SEM) model

In this study, we set six hypotheses to investigate whether COVID-19 risk becomes biased among individuals with different political preferences. We hypothesised that an individual’s political orientation serves as a crucial factor influencing trust in the government, particularly in its role in formulating policies to mitigate and respond to the COVID-19 risk. Existing research observed that people tended to vary in their support for government policies aimed at mitigating hazards, depending on their political orientation [35]. Thus, this study posited that individuals who trust government policies are more likely to engage in preventive behaviour and adhere to public compliance, aligning with findings from COVID-19 cases in the USA [20,36]. Given that individuals’ behaviours against risks are inevitably influenced by their risk perception [37], a conceptual model was designed to elucidate that trust in government policies indirectly affects engagement in preventive behaviour through individuals’ risk perception.

Building upon these principles, the following six hypotheses were proposed: 1) individuals whose political orientation aligns with that of the government are more likely to trust its response to mitigating the COVID-19 pandemic, 2) individuals whose political orientation aligns with that of the government are more likely to perceive the risk of COVID-19, 3) individuals who trust the government are more likely to engage in preventive behaviour, 4) individuals whose political orientation aligns with that of the government are more likely to engage in preventive behaviour, 5) individuals who trust the government are less likely to perceive the risk of COVID-19, and 6) individuals with risk perception are more likely to engage in preventive behaviour.

**Figure 1:**
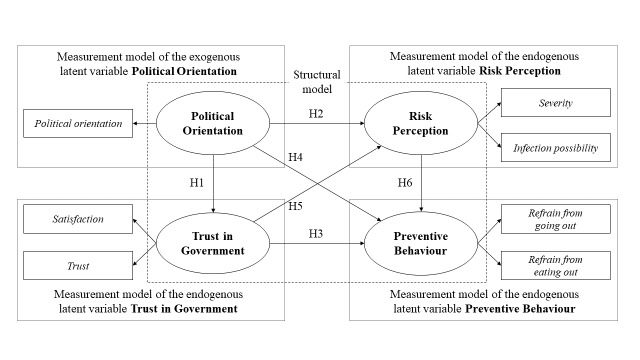
SEM for examining politically constructed COVID-19 risk perception and preventive behaviour. COVID-19 – coronavirus disease 2019.

We employed a SEM to investigate the six hypotheses. Especially, we adopted a PLS-based SEM to realise the conceptual model (**Figure 1**). We used the PLS-SEM, a variance-based SEM method, to discern the causal relationships among latent variables and to estimate the path coefficients within each hypothesis [38]. It comprises two sets of linear models – the measurement and structural models. The measurement model establishes the relationship between a latent variable and its exploratory variables, while the structural model aims to illustrate the relationship among latent variables. We organised the measurement models into four latent variables – political orientation, trust in government, risk perception, and preventive behaviour, constructed using seven exploratory variables. Two latent variables, trust in government and risk perception, operate as mediator variables between political orientation and preventive behaviour. We carefully chose relevant exploratory variables from the survey questionnaires for each latent variable, ensuring that the selection meets the criteria for model and measurement fit.

We focused on examining the two PLS-SEM results of the Delta variant during the Moon Jae-in administration and the Omicron variant during the Yoon Suk-yeol administration. During the Moon Jae-in administration, we utilised a 38th survey (30 July–2 August 2021) to capture the moments associated with the Delta variant. After the shift from a progressive to a conservative government regime, we employed the 62nd survey conducted from 25–28 February 2022 to investigate the six hypotheses amid the emergence of the Omicron variant. After excluding non-responsive and irrelevant samples, such as missing responses, the final sample sizes utilised were 913 at the 38th and 871 at the 62nd survey. Utilising the results from the two cross-sectional surveys, we investigated the influence of political orientation on individuals’ risk perception and intention to engage in preventive behaviour, both of which were compared at two distinct moments characterised by politically opposite tendencies in government regimes.

For the cross-sectional analyses, we constructed the PLS-SEM using Smart-PLS4 (SmartPLS GmbH, Boenningstedt, Germany), which has been widely utilised for analysing PLS-SEM [38–42]. As the PLS-SEM does not assume normal distribution of data, we adopted a non-parametric bootstrap technique to identify statistically significant paths. The bootstrapping method involves the creation of subsamples randomly selected from the original data, and these subsamples are then utilised to estimate the path coefficients. This study created 10 000 subsamples, which has been deemed a sufficient quantity [41], to derive parameter estimates with 95% confidence intervals for significance testing. We visualised the relationship between each latent variable through path coefficients (β) and a corresponding significance level at 99%.

## RESULTS

### Temporal trends of risk perception and preventive behaviour

**Figure 2:**
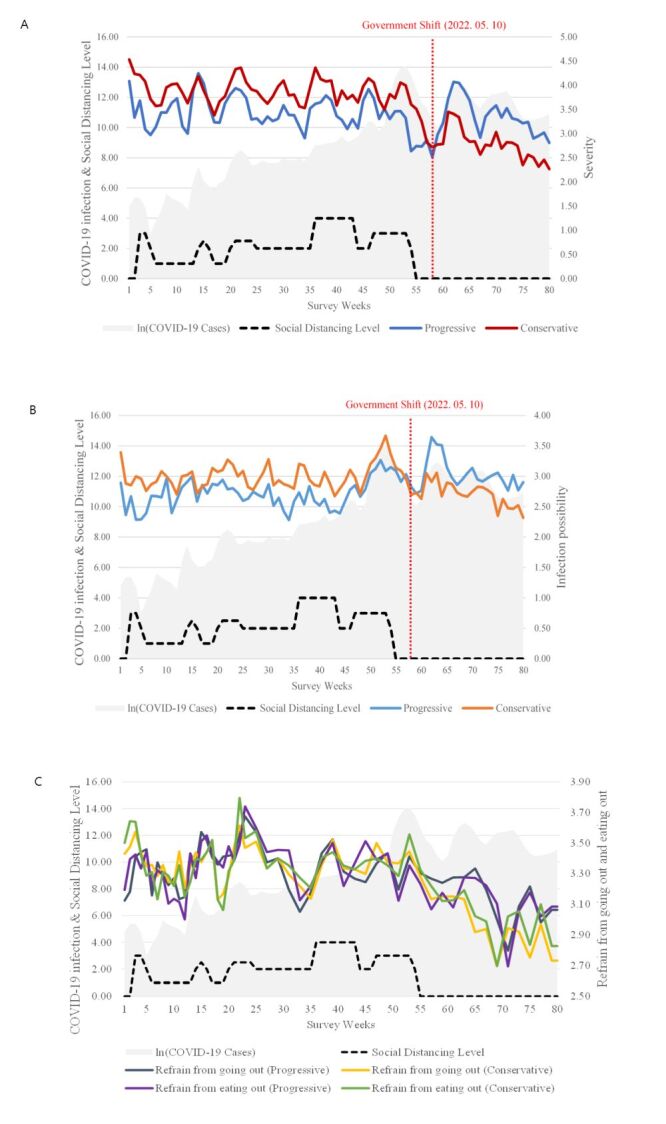
Risk perception and behavioural intention of politically progressive and conservative groups. **Panel A.** Perceived COVID-19 severity. **Panel B.** Perceived COVID-19 infection possibility. **Panel C.** Intention to refrain from going out and eating out. COVID-19 – coronavirus disease 2019.

During the survey period, we illustrated the biweekly trends in the risk perception of severity and infection possibility, as perceived by politically progressive and conservative groups (**Figure 2**, Panel A–B). We observed a dramatic change in the overall trend of risk perception following the government’s transition from the Moon Jae-in to the Yoon Suk-yeol administration. Progressive groups exhibited a decreasing pattern in their perceived severity of COVID-19 spread before the government shift. However, immediately after the conservative administration took over, their response to the same question became more negative, indicating that they considered the spread of COVID-19 to be serious.

By contrast, conservative groups perceived the Severity of COVID-19 spread to be slightly higher than that of progressive groups during the progressive administration period. However, after the government shift, their perception significantly decreased, falling below that of progressive groups. Although there are differences in degree, perceived COVID-19 Infection possibility also showed similar trends with Severity among political orientation groups, both before and after the shift.

Survey responses detailing the refrain from going out and refrain from eating out of both progressive and conservative groups are illustrated in **Figure 2**, Panel C. Unlike risk perception, preventive behaviour demonstrated no significant shifts in responses between the progressive and conservative groups, irrespective of government tendency. The findings from these two aspects suggest that individuals’ COVID-19 risk perception was susceptible to influence from their political orientation, whereas their actual behaviour seemed to be more responsive to other factors such as the fatality of COVID-19 or the government’s social distancing levels [43].

### Cross-sectional analysis (PLS-SEM)

We adopted various statistical parameters to validate the structural model in PLS-SEM based on the criteria proposed by previous studies [38–42]. Initially, we evaluated the sufficiency of the sample size for building the PLS-SEM model. It is recommended that the number of samples is at least 10 times larger than the maximum number of paths directed towards any individual latent variable in the PLS-SEM model [39]. The model depicted in **Figure 1** includes three paths directed towards prevention behaviour, indicating that the sample size in each survey is sufficient to meet the minimum requirement of 30 samples. Second, we examined whether the overall model fit for each PLS-SEM meets the specified criteria. We utilised four parameters, including the standardised root mean square residual, the squared Euclidean distance, the geodesic distance, and the normed fit index, based on the previous studies [38–42], to assess model fit and confirmed that the obtained values fall within the recommended ranges (Table S1 in the **Online Supplementary Document**).

In the measurement model of PLS-SEM across two different administrations, this study conducted multiple tests to confirm internal consistency reliability, convergent validity, and discriminant validity [38–42]. The internal consistency reliability can be assessed through measurements such as Cronbach’s α and composite reliability, which indicate the explanatory power of the manifest variables in relation to the corresponding latent variable. All latent variables, excluding risk perception, satisfied the criterion for internal consistency reliability, with both values exceeding 0.7 in two different administrations (Table S2 in the **Online Supplementary Document**). Considering that the convergent validity is confirmed when the average variance extracted (AVE) value exceeds 0.5, all latent variables without risk perception surpassed this minimum criterion. The rationale for not meeting the requirement in risk perception can be attributed to the relatively lower cross-loading score of infection possibility compared with severity in the latent variable of risk perception. While severity exhibited a score over 0.7, infection possibility obtained a lower value, falling below 0.7. Nevertheless, we included the infection possibility as a manifest variable due to its second-highest cross-loading score among others.

We evaluated discriminant validity through cross-loadings between each manifest variable to assess the relevance of these variables to the target latent variable (Table S3 in the **Online Supplementary Document**). Each latent variable effectively represented its manifest variables, as evidenced by their higher cross-loading scores compared with the others. For example, the cross-loadings of satisfaction and trust displayed higher scores in trust in government compared with the other latent variables. Severity and infection possibility exhibited relatively high correlations with risk perception, while refraining from going out and refraining from eating out showed high cross-loadings in preventive behaviour across the two distinct administrations.

**Figure 3:**
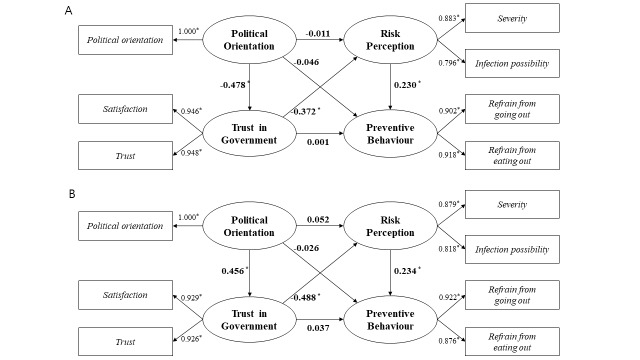
Cross-sectional results derived by PLS-SEM *P < 0.01. **Panel A.** Amid the spread of the Delta variant under the Moon Jae-in administration. **Panel B.** Amid the spread of the Omicron variant under the Yoon Suk-yeol administration.

The results of the PLS-SEM provided in-depth evidence that individuals’ risk perception was socially constructed based on their political orientation. Three hypotheses (hypotheses one, five, and six) were statistically significant under both administrations (**Figure 3**, Panels A–B, **Table 2**). As the pandemic progressed, the cross-sectional results revealed a notable trend wherein Koreans perceived the risk of COVID-19 as a political issue rather than an objective public health threat. This politicisation of risk perception was most clearly reflected in the first hypothesis, which demonstrated a marked contrast across administrations. Under the Moon Jae-in administration, individuals with more conservative political leanings were significantly less likely to trust the government’s pandemic response (β = –0.478, *P* < 0.01). In contrast, during the Yoon Suk-yeol administration, led by the conservative party, this relationship reversed (β = 0.456, *P* < 0.01), with conservatives expressing greater trust in the government's handling of the pandemic.

**Table 2:** Results of path coefficients with a *P*-value for the PLS-SEM at the Delta and Omicron variants

	Moon Jae-in administration (progressive-oriented)		Yoon Suk-yeol administration (conservative-oriented)	
**Hypothesis**	**Path coefficient (β)**	***P*-value**	**Path coefficient (β)**	***P*-value**
H1: Political Orientation → Trust in Government	–0.478	0.000	0.456	0.000
H2: Political Orientation → Risk Perception	–0.011	0.784	0.052	0.165
H3: Trust in Government → Preventive Behaviour	0.001	0.983	0.037	0.407
H4: Political Orientation → Preventive Behaviour	–0.046	0.274	–0.026	0.537
H5: Trust in Government → Risk Perception	–0.372	0.000	–0.488	0.000
H6: Risk Perception → Preventive Behaviour	0.230	0.000	0.234	0.000

PLS-SEM – partial least squares structural equation modelling

Despite the different political tendencies between the two administrations, a consistent correlation in the paths was observed between trust in government and risk perception (fifth hypothesis), as well as between risk perception and preventive behaviour (sixth hypothesis) (*P* < 0.01). Individuals who trusted the government and supported its policies were more likely to exhibit lower risk perception. The intention to engage in preventive behaviour was positively influenced by the extent of risk perception.

In conclusion, individuals whose political orientation differed from that of the ruling administration tended to exhibit lower levels of trust in the government’s pandemic response, which in turn led to heightened risk perception. This elevated sense of risk subsequently had a positive impact on individuals’ engagement in preventive behaviours. Notably, behavioural intention was not directly shaped by political orientation or trust in government; rather, it was indirectly influenced through the mediating role of politically constructed risk perception.

## DISCUSSION

At the onset of the COVID-19 pandemic, a tendency for individuals opposing the ruling party to exhibit lower risk perception and reduced confidence in government policies was observed across countries with differing political administrations, as seen in the USA and South Korea [16,19]. While economic factors and personal experiences also significantly influenced pandemic risk perception and related behaviours [44–46], this research specifically examines the impact of political preference on risk perception and preventive behaviour by analysing 80 waves of repeated cross-sectional survey data from South Korea across the full course of the pandemic.

Temporal trends analysis of risk perception offered a clear observation that individuals with a political orientation opposite to that of the government tended to exhibit higher levels of risk perception regardless of government regime shift. This finding implies that respondents’ political orientation and the ruling party have a significant and persistent impact on their risk perception throughout the pandemic. While we identified a distinct pattern of risk perception among politically different groups, we did not uncover a corresponding pattern in behavioural intention to engage in preventive measures against COVID-19. For a comprehensive analysis of political biases associated with the risk of COVID-19, we employed a cross-sectional method to identify the factors influencing risk perception and behavioural intention.

The cross-sectional analysis provides in-depth evidence of the political influence on individuals’ risk perception and intention to engage in preventive behaviours. Individuals’ political preferences were closely associated with their trust in the government’s responses, and risk perception was configured differently depending on the level of government trust. Notably, these relationships persisted even after the regime transitioned to a conservative administration, despite minimal change in the government’s quarantine policies.

This suggests that individuals whose political stance was aligned with that of the government were more likely to trust its response to the pandemic. In other words, individuals’ trust in the government is more likely influenced by their political stance relative to the ruling party rather than by the objective risk or the government’s quarantine policies. Consequently, individuals with lower trust in government policies are likely to have an elevated risk perception, which subsequently leads to increased adoption of preventive behaviours. Ultimately, Koreans’ risk perception during the COVID-19 pandemic appears to have been more politically influenced than directly linked to the objective risk.

The results of this study can be interpreted as a manifestation of political polarisation that is prone to occur in countries with a deeply entrenched two-party political system, such as South Korea, where partisan divisions are often pronounced. Political polarisation, a growing concern worldwide with significant negative impacts on various national issues, including the environment and public health, can manifest as intergroup polarisation. In the context of Korea’s COVID-19 response, this intergroup polarisation is particularly evident as it appears driven by shifts in administration rather than concrete policy changes. Unlike opinion polarisation, intergroup polarisation centres on the formation of ideological ingroups and outgroups [47–49]. This often involves experiencing negative emotions like hatred towards perceived outgroups and positive attitudes towards one's ingroup, such as a preferred political party [47–49].

Given that politically motivated risk divergence stems from a deeper emphasis on personal identity or group affiliation, conventional methods such as logical argumentation or direct persuasion often prove ineffective. These approaches can inadvertently fuel polarisation by creating an ‘us *vs*. them’ mentality [49], a key characteristic of intergroup polarisation discussed above. Instead, communication strategies should prioritise bridge-building approaches. In such strategies, leaders or mediators position themselves in the middle ground, actively listen to diverse perspectives, and foster mutual recognition. These ‘bridge builders’ play a crucial role in calming tensions and presenting shared visions that can reconnect polarised groups.

Based on characteristics of polarisation, public health agencies have placed an emphasis on adopting inclusive and identity-sensitive communication with shared values such as safety, community well-being, and mutual responsibility. Appointing neutral or bipartisan spokespersons can enhance perceived credibility across partisan lines. Furthermore, fostering deliberative spaces for intergroup dialogue – where opposing views can be aired in a respectful and moderated environment – may reduce mistrust. Agencies should also remain aware of the current conflict stage in society and adjust their outreach accordingly, moving from prevention and engagement to reconciliation as necessary. By refraining from separating people into two opposing groups, we can ultimately achieve trust by building social cohesion and dialogue.

## CONCLUSIONS

In this study, we highlight how individuals’ perceptions of COVID-19 risk were shaped by political preferences, illustrating that the pandemic response became a subject of political division not only between two hostile political parties but also among citizens. Rather than being viewed purely as a public health threat, COVID-19 was increasingly perceived through a politicised lens influenced by partisan bias. Individuals exhibit different perspectives on COVID-19 based on their political support for specific political parties, shaping their risk perception during the pandemic. Especially, trust in the government is a pivotal determinant of individuals’ risk perception. Those whose political orientation is aligned with that of the government are inclined to trust its countermeasures against COVID-19, leading to lower levels of risk perception compared to individuals holding opposing political views. While this study did not account for socio-demographic traits in the analysis, the conclusion that COVID-19 has become politicised remains valid, as it is grounded in survey responses representative of the general Korean population.

The results of this study hold important implications for future practice. We identified that risk can be socially constructed, particularly in terms of political ingroups and outgroups. Future research should endeavour to formulate quarantine and health policies that understand the characteristics of risk perceptions. In essence, individuals’ risk perception is not solely grounded in actual risk but is also socially constructed, particularly within a political context. Therefore, when striving for collaborative problem-solving, as witnessed during the COVID-19 pandemic, it is important to caution against the politicisation of the issues. These politicised issues may lead to prejudicial criticism of opposing parties, hinder efforts to seek common values, discourage intergroup communication through diverse media, and ultimately undermine public engagement in the processes of deliberative democracy.

While we primarily focused on the political dimension, we acknowledge the significant potential influence of economic factors and personal experiences with COVID-19 on risk perception [44–46]. Future research could explore the interplay of these factors alongside political orientation, examining how economic anxieties (*e.g.* job security, financial strain) might shape individuals' risk assessments and adherence to public health measures. Similarly, future studies could investigate how direct personal experiences with COVID-19, such as contracting the virus oneself or witnessing its impact on loved ones, might influence risk perception, potentially overriding or interacting with political leanings.

## Additional material


Online Supplementary Document
