# Supplementary material for: Political construction of risk perception and preventive behaviour during the COVID-19 pandemic in the Republic of Korea
Source: J Glob Health. 2025 Jul 4;15:04189. doi: 10.7189/jogh.15.04189 (PMC12231513; doi:10.7189/jogh.15.04189)
Supplement: Online Supplementary Document [file jogh-15-04189-s001.pdf]

Supplement to: Jeong S, Chung JB, Kim M, Kim MK. Political construction of risk perception and preventive behaviour during the COVID-19 pandemic in the Republic of Korea. J Glob Health. 2025;15:04189.

Table S1. Results of overall and measurement fit and cross-loadings for the PLS-SEM – overall model fit

Statistic	Recommended value	Obtained value	
		Moon Jae-in administration (Progressive-oriented)	Yoon Suk-yeol administration (Conservative-oriented)
SRMR	< 0.1	0.018	0.023
d_ULS	< 0.95	0.009	0.014
d_G	< 0.95	0.003	0.011
NFI	> 0.9	0.992	0.972

d_G – geodesic distance, d_ULS – squared Euclidean distance, NFI – normed fit index, SRMR – standardized root mean square residual

Table S2. Results of overall and measurement fit and cross-loadings for the PLS-SEM – measurement fit

Latent variable	Manifest variable	Moon Jae-in administration (Progressive-oriented)			Yoon Suk-yeol administration (Conservative-oriented)		
		Cronbach's α	CR	AVE	Cronbach's α	CR	AVE
Political Orientation	*Political orientation*	1.000	1.000	1.000	1.000	1.000	1.000
Trust in Government	*Satisfaction*<br>*Trust*	0.884	0.884	0.792	0.837	0.837	0.720
Risk Perception	*Severity*<br>*Infection possibility*	0.590	0.614	0.432	0.616	0.631	0.453
Preventive Behavior	*Refrain from going out*<br>*Refrain from eating out*	0.792	0.796	0.658	0.766	0.793	0.637

AVE – average variance extracted, CR – composite reliability

Table S3. Results of overall and measurement fit and cross-loadings for the PLS-SEM – cross-loadings for the PLS-SEM

Manifest variable	Moon Jae-in administration (Progressive-oriented)				Yoon Suk-yeol administration (Conservative-oriented)			
	Political Orientation	Trust in Government	Risk Perception	Preventive Behavior	Political Orientation	Trust in Government	Risk Perception	Preventive Behavior
Political orientation	**1.000**	-0.508	0.213	-0.009	**1.000**	0.499	-0.215	-0.056
Satisfaction	-0.459	**0.882**	-0.426	-0.034	0.379	**0.855**	-0.407	-0.082
Trust	-0.445	**0.898**	-0.46	-0.096	0.469	**0.842**	-0.476	-0.092
Severity	0.137	-0.349	**0.733**	0.261	-0.166	-0.471	**0.733**	0.225
Infection possibility	0.146	-0.305	**0.57**	0.145	-0.121	-0.384	**0.608**	0.196
Refrain from going out	0.003	-0.066	0.249	**0.777**	-0.051	-0.11	0.279	**0.881**
Refrain from eating out	-0.017	-0.053	0.265	**0.844**	-0.036	-0.047	0.216	**0.705**
